# Effects of Capping Layers with Different Metals on Electrical Performance and Stability of p-Channel SnO Thin-Film Transistors

**DOI:** 10.3390/mi11100917

**Published:** 2020-09-30

**Authors:** Min-Gyu Shin, Kang-Hwan Bae, Hwan-Seok Jeong, Dae-Hwan Kim, Hyun-Seok Cha, Hyuck-In Kwon

**Affiliations:** School of Electrical and Electronics Engineering, Chung-Ang University, Seoul 06974, Korea; 18alsrb@naver.com (M.-G.S.); rkdghks95@naver.com (K.-H.B.); hwanseok518@cau.ac.kr (H.-S.J.); pccdhkim@naver.com (D.-H.K.); ckgustjr0803@naver.com (H.-S.C.)

**Keywords:** SnO, p-channel thin-film transistor, metal capping layer, work function, electrical performance and stability

## Abstract

In this study, the effects of capping layers with different metals on the electrical performance and stability of p-channel SnO thin-film transistors (TFTs) were examined. Ni- or Pt-capped SnO TFTs exhibit a higher field-effect mobility (*μ*_FE_), a lower subthreshold swing (*SS*), a positively shifted threshold voltage (*V*_TH_), and an improved negative-gate-bias-stress (NGBS) stability, as compared to pristine TFTs. In contrast, Al-capped SnO TFTs exhibit a lower *μ*_FE_, higher *SS*, negatively shifted *V*_TH_, and degraded NGBS stability, as compared to pristine TFTs. No significant difference was observed between the electrical performance of the Cr-capped SnO TFT and that of the pristine SnO TFT. The obtained results were primarily explained based on the change in the back-channel potential of the SnO TFT that was caused by the difference in work functions between the SnO and various metals. This study shows that capping layers with different metals can be practically employed to modulate the electrical characteristics of p-channel SnO TFTs.

## 1. Introduction

Since the first report on indium-gallium-zinc oxide thin-film transistors (TFTs) by Nomura et al. in 2004, oxide semiconductor TFTs have drawn significant attention owing to their high electrical performance, low fabrication temperature, and superior large area uniformity [1,2,3,4,5,6,7,8,9,10]. To date, a variety of oxide semiconductors have been developed to enhance the electrical performance and stability of oxide TFTs [11,12,13,14,15,16,17,18,19,20]. However, fabricating p-channel oxide TFTs consisting of electrical performance comparable to that of n-channel oxide TFTs remain a challenge. Complementary logic circuits using both n- and p-channel field-effect transistors (FETs) have several advantages, such as higher noise margin, lower power consumption, higher integration density, higher gain, and simpler biasing over electronic circuits consisting of only n-channel FETs [21,22,23,24,25,26,27,28]. Therefore, it is important to improve the electrical performance and stability of p-channel oxide TFTs to expand the application area of oxide TFTs to more sophisticated electronic systems.

Among various p-type oxide semiconductors, tin monoxide (SnO) has attracted significant attention as a channel material for p-channel oxide TFTs. This is because the hybridization of pseudo-closed Sn 5s and O 2p orbitals in SnO form delocalized and isotropic orbitals near the valence-band maximum (VBM), unlike in other p-type oxide semiconductors. This VBM structure provides an effective hole conduction path, thereby making SnO a potential candidate for the channel material of high mobility p-channel oxide TFTs [29,30,31,32,33]. In our previous report, we demonstrated that the formation of a Ni capping layer can increase the field-effect mobility (*μ*_FE_) of p-channel SnO TFTs. This was attributed to the changed vertical electric field distribution and increase in the hole concentration inside SnO [34]. In this study, we compared the electrical performance and stability of p-channel SnO TFTs consisting of capping layers with different metals (Al, Ni, Pt, and Cr) to thoroughly understand the effects of a metal capping layer on p-channel SnO TFTs. Our experimental results indicate that the electrical performance and stability of p-channel SnO TFTs significantly change depending on the metal used for the capping layers. This implies that the metal capping layer can be practically used to modulate the electrical characteristics of p-channel SnO TFTs. A systematic study was conducted to reveal the physical mechanisms responsible for the observed phenomena.

## 2. Experimental Procedure

**Figure 1:**
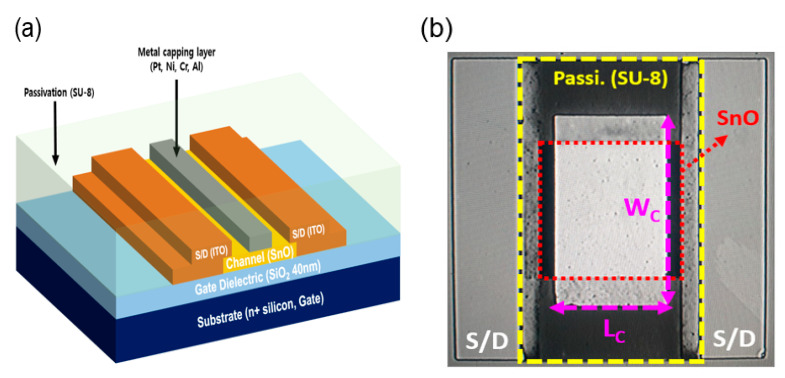
(**a**) Cross-sectional schematic view; (**b**) optical image of the fabricated p-channel SnO thin-film transistors (TFT) with a metal capping layer.

Bottom-gate top-source/drain electrode TFTs were fabricated on thermally oxidized SiO_2_/n^+^-silicon substrates. The thermal SiO_2_ layer had a thickness of 40 nm. All patterns were defined using the lift-off process. A 16-nm-thick SnO_X_ thin film was formed via reactive radio frequency sputtering using a metallic Sn target (3-in-diameter, 99.999%) without heating. During the deposition, the Ar/O_2_ ratio was 90:4 (sccm:sccm), RF power was fixed at 60 W, and deposition pressure was 3 mTorr. Before forming the source/drain electrodes, the SnO_X_ thin film was annealed in air at 180 °C for 30 min using a hot plate [35]. The source and drain were formed using 100-nm-thick indium-tin oxide (ITO), which was deposited via direct current magnetron sputtering. Sequentially, 50-nm-thick capping layers with different metals (Al, Ni, Pt, and Cr) were formed on top of the patterned SnO_X_ thin films using an e-gun evaporator under a high vacuum of 5 × 10^−6^ Torr. Finally, a passivation layer was formed on the fabricated TFTs using a 2-μm-thick spin-coated SU-8. Figure 1a,b show the schematic view and optical image of the fabricated metal-capped TFTs, respectively. The channel width and length) were both 500 μm. The width and length of the metal capping layers were 700 μm and 400 μm, respectively. The surface morphologies of the SnO_X_ thin films were obtained via field-emission scanning electron microscopy (FE-SEM, JEOL, Boston, MA, USA) and atomic force microscopy (AFM, Park Systems, Suwon, Korea). The transmission spectra of the thin films were obtained using a spectrophotometer in the ultraviolet visible-near infrared (UV-vis-NIR, JASCO, Pfungstadt, Germany, wavelength: 300–1400 nm) region. The structural properties of the tin oxide thin films were characterized by X-ray diffraction (XRD, Rigaku, Tokyo, Japan) in the *θ*–2*θ* mode with Cu-Kα radiation (λ = 0.15418 nm). The work-functions (*Φ*_S_) of deposited SnO_X_ thin film and capping metal layers were determined using the Kelvin probe force microscopy method (KP Technology, Scotland, UK). The electrical characteristics were measured using an Agilent 4156C precision semiconductor parameter analyzer (Agilent Technologies, Santa Clara, CA, USA) in the dark at room temperature in ambient air.

## 3. Results and Discussion

**Figure 2:**
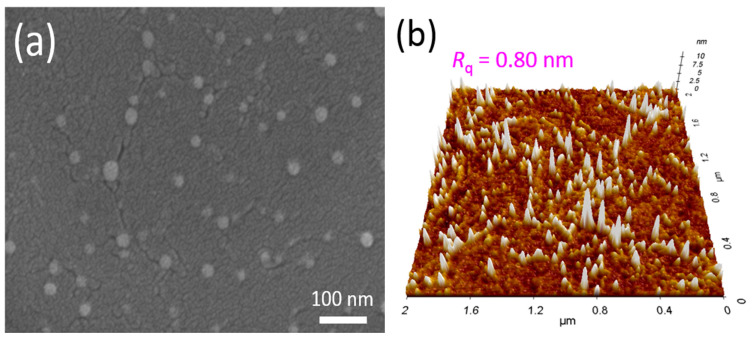
(**a**) Field-emission scanning electron microscopy (FE-SEM); (**b**) atomic force microscopy (AFM) images of the 16-nm-thick SnO_X_ thin film deposited on the thermally oxidized SiO_2_/n^+^-silicon substrate.

**Figure 3:**
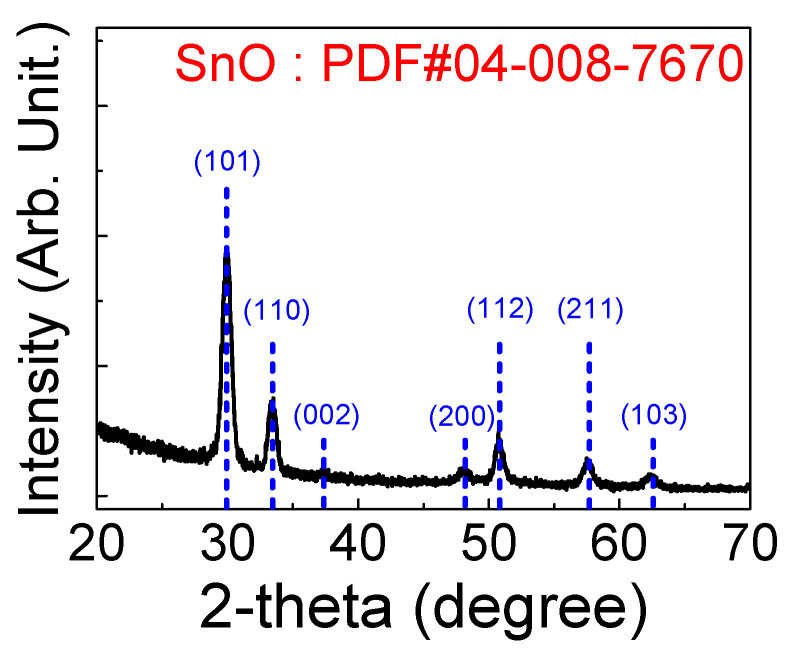
X-ray diffraction (XRD) patterns of the SnO_X_ thin film deposited on the thermally oxidized SiO_2_/n^+^-silicon substrate.

**Figure 4:**
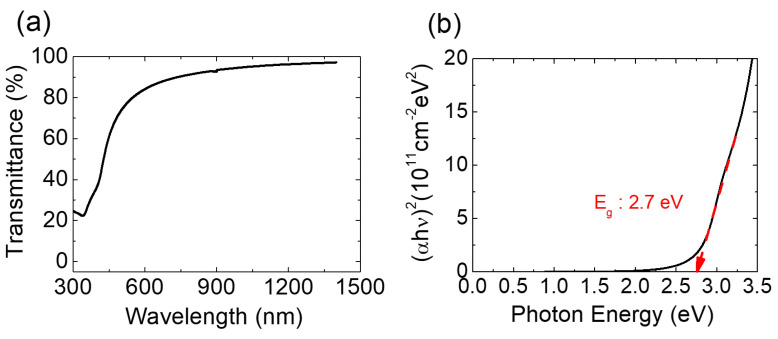
(**a**) Optical transmittance spectra of the SnO_X_ thin film; (**b**) Tauc plot calculated from the measured optical transmittance spectra.

Figure 2a,b display the FE-SEM and AFM images of the 16-nm-thick SnO_X_ thin film deposited on the thermally oxidized SiO_2_/n^+^-silicon substrate. Figure 2a shows the polycrystalline microstructure of SnO_X_ thin film containing irregularly-shaped grains. Strip-wise-cracks are considered the grain boundaries, and the bright spots are speculated to be the metallic Sn particles [36]. Figure 2b shows that the root mean square roughness (*R*_q_) of the deposited SnO_X_ thin film is approximately 0.80 nm. Figure 3 presents the XRD patterns of the 16-nm-thick SnO_X_ thin film deposited on the thermally oxidized SiO_2_/n^+^-silicon substrate. Diffraction peaks are observed from the XRD patterns, implying that the deposited SnO_X_ thin film has a polycrystalline phase. The sharp peaks corresponding to the (002), (101), (103), (110), (112), (200), and (211) planes [37] of the SnO phase are observed, which indicates that SnO is the dominant phase in the deposited thin film. Figure 4a displays the optical transmittance spectra of the SnO_X_ thin film. The optical transmittance spectra were measured using the SnO_X_ thin film deposited on glass (Corning Eagle XG) within the wavelength range of 300–1400 nm. The optical transmittance of the glass was removed from the measured data to obtain accurate optical transmittance values of the deposited SnO_X_ thin film. Figure 4b shows the Tauc plot calculated from Figure 4a. The optical bandgap (*E*_g_) of the deposited thin film was obtained as 2.7 eV, which is consistent with the reported values for SnO in previous studies [38]. From the Kelvin probe force microscopy, the *Φ* of the deposited SnO thin film was determined to be 4.6 eV. The *Φ* value was measured using cantilevers composed of Au, whose work function was determined by calibration with reference sample Au prepared in ultrahigh vacuum condition.

**Figure 5:**
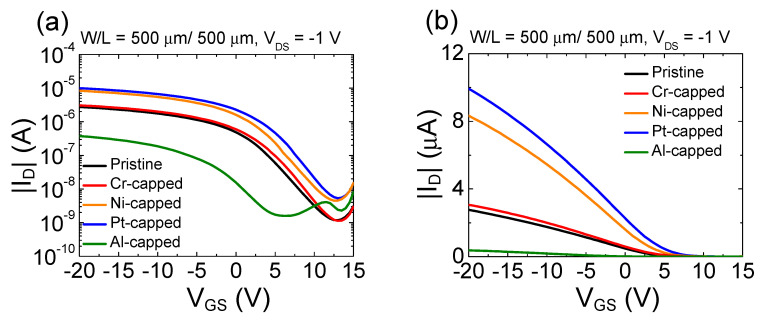
(**a**) Representative transfer curves of the pristine SnO thin-film transistors (TFTs) and the Al-, Ni-, Pt-, and Cr-capped SnO TFTs in the semi-logarithmic scale; (**b**) representative transfer curves of the pristine SnO TFTs and the Al-, Ni-, Pt-, and Cr-capped SnO TFTs in the linear scale.

Figure 5a,b present the representative transfer curves of the pristine and Al-, Ni-, Pt-, and Cr-capped SnO TFTs in the semi-logarithmic and linear scale, respectively, where *V*_GS_, *V*_DS_, and *I*_D_ are the gate-to-source voltage, drain-to-source voltage, and drain current, respectively. Measurements were conducted by scanning *V*_GS_ from 15 to −20 V at *V*_DS_ = −1.0 V for all SnO TFTs. Figure 5 indicates that the transfer curve of the Cr-capped SnO TFT resembles that of the pristine SnO TFT. However, compared to the pristine SnO TFT, a significant change in the shape of the transfer curve is observed from the SnO TFTs capped with other metals. Table 1 lists the electrical parameters extracted from the SnO TFTs capped with different metals, where the threshold voltage (*V*_TH_) is determined by finding the intercept of the linearly extrapolated linear scale transfer curve with the *V*_GS_ axis and *μ*_FE_ is extracted from the maximum transconductance at a *V*_DS_ of −1.0 V. *I*_ON/OFF_ is the current on-off ratio. The subthreshold swing (*SS*) value was determined from the semi-logarithmic scale transfer curve using Equation (1).

(1) $$SS=\frac{dV_{\mathrm{GS}}}{d\left(\log I_{D}\right)}$$

The extracted data in Table 1 indicate that *V*_TH_, *μ*_FE_, and *SS* of the SnO TFT have significantly different values depending on the metal utilized for the formation of the capping layer. The Al-capped SnO TFT exhibits a lower value of *μ*_FE_ (0.3 cm^2^/V·s), higher value of *SS* (4.7 V/decade), and negatively shifted *V*_TH_ (−1.6 V) than the pristine SnO TFT (*μ*_FE_ = 1.6 cm^2^/V·s, *SS* = 4.0 V/decade, and *V*_TH_ = 3.7 V). In contrast, the Ni- and Pt-capped SnO TFTs exhibit a higher value of *μ*_FE_ (4.8 cm^2^/V·s and 5.4 cm^2^/V·s for Ni- and Pt-capped TFTs, respectively), lower value of *SS* (3.7 V/decade and 3.5 V/decade for Ni- and Pt-capped TFTs, respectively), and positively shifted *V*_TH_ (5.2 V and 6.3 V for Ni- and Pt-capped TFTs, respectively) than the pristine device. No significant difference was observed between the electrical properties of the Cr-capped SnO TFT (*μ*_FE_ = 1.8 cm^2^/V⋅s, *SS* = 3.9 V/decade, and *V*_TH_ = 4.5 V) and the pristine SnO TFT. Therefore, the results in Figure 5 and Table 1 demonstrate that using different metals in the formation of capping layers can effectively modulate the electrical characteristics of p-channel SnO TFTs.

**Figure 6:**
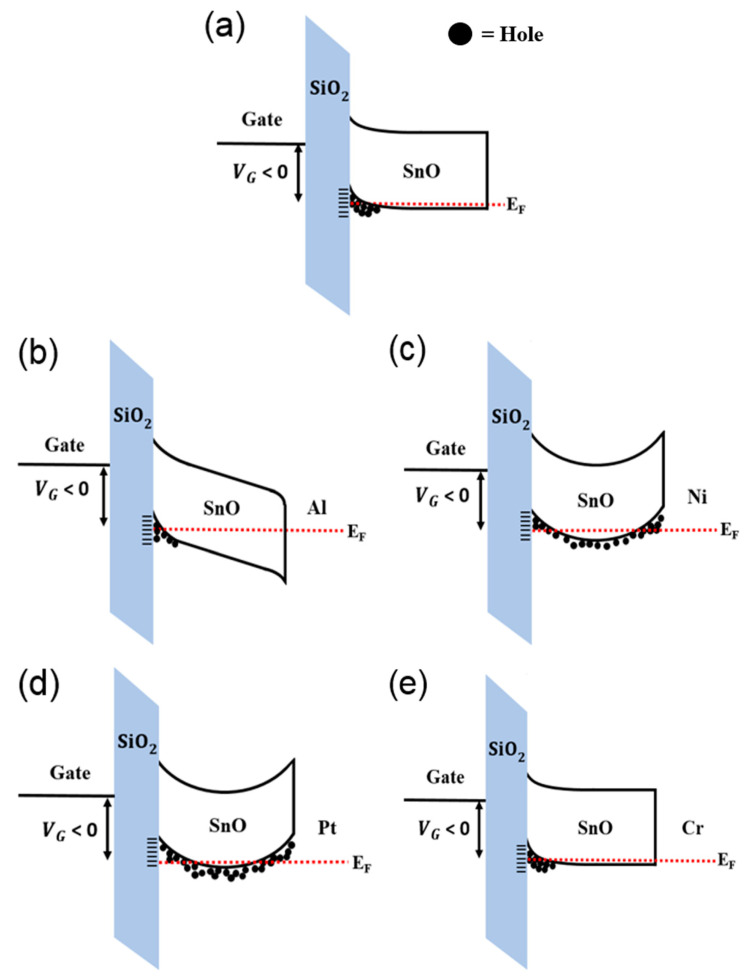
Energy band diagrams for (**a**) pristine, (**b**) Al-capped, (**c**) Ni-capped, (**d**) Pt-capped, and (**e**) Cr-capped SnO channels under a negative *V*_GS_ at *V*_DS_ = 0 V.

The most plausible reason for the observed phenomena in Figure 5 is the change in the back-channel potential of the SnO TFT caused by the difference of *Φ* between the SnO and different species of metals. Figure 6a,e display the schematic energy band diagrams for pristine and Al-, Ni-, Pt-, and Cr-capped SnO channels under an application of negative *V*_GS_. Figure 6b depicts the schematic energy band diagram for the Al-capped SnO channel. Al has a *Φ* of 4.1 eV [39,40], which is lower than that of the SnO (*Φ* = 4.6 eV). Therefore, the energy band near the back-channel surface displays downward bending, thereby forming the depletion region near the SnO-Al interface. The decreased hole concentration inside the SnO channel shifts the *V*_TH_ of the SnO TFT in the negative direction. In addition, contrary to the pristine TFT, the depletion region formed near the back-channel surface in the Al-capped SnO TFT forces the holes to move closer to the SiO_2_/SnO interface, which can degrade the *μ*_FE_ value of the SnO TFTs because of the increased surface roughness scattering.Another possible mechanism for the decrease of the *μ*_FE_ value in the Al-capped SnO TFT is the lowered percolation conduction probability caused by the decreased hole concentration inside the SnO channel. Previous studies reported that the randomly distributed Sn^2+^ ions generate an energy distribution barrier near the VBM, and the percolation conduction has been considered one of the dominant carrier transport mechanisms in SnO TFTs [41]. 

Figure 6c,d depict the schematic energy band diagrams for the Ni- and Pt-capped SnO channels, respectively. Ni and Pt have a *Φ* of 5.2 (Ni) [42,43] and 5.6 (Pt) eV [44,45], which are higher than that of the SnO. As reported in our previous study, the capping metals with higher *Φ* than SnO accumulate holes in the upward bending of the energy band at the SnO−Ni/Pt interface [27]. An increased hole concentration inside the SnO channel shifts the *V*_TH_ of the SnO TFT in the positive direction and enhances the percolation conduction probability and *μ*_FE_. In addition, the upward bending of the energy band can form the bulk accumulation channel during an application of the negative *V*_GS_, as demonstrated in Figure 6c,d, owing to the thickness of the thin film (16 nm). Formation of the bulk accumulation channel can be another reason for the increased *μ*_FE_ because holes can avoid scattering at the interface when they cross the bulk accumulation channel [46]. That Pt-capped SnO has higher values of *μ*_FE_ and more positively shifted *V*_TH_ than the Ni-capped device can be attributed to the higher *Φ* value of Pt than that of Ni. Figure 6e depicts the schematic energy band for the Cr-capped SnO channel. Cr has a similar value of *Φ* to SnO [47,48]; therefore, the band bending at the back channel of SnO is expected to be insignificant. This is considered to be the cause of the nearly identical electrical properties of Cr-capped SnO TFT and the pristine TFT.

**Figure 7:**
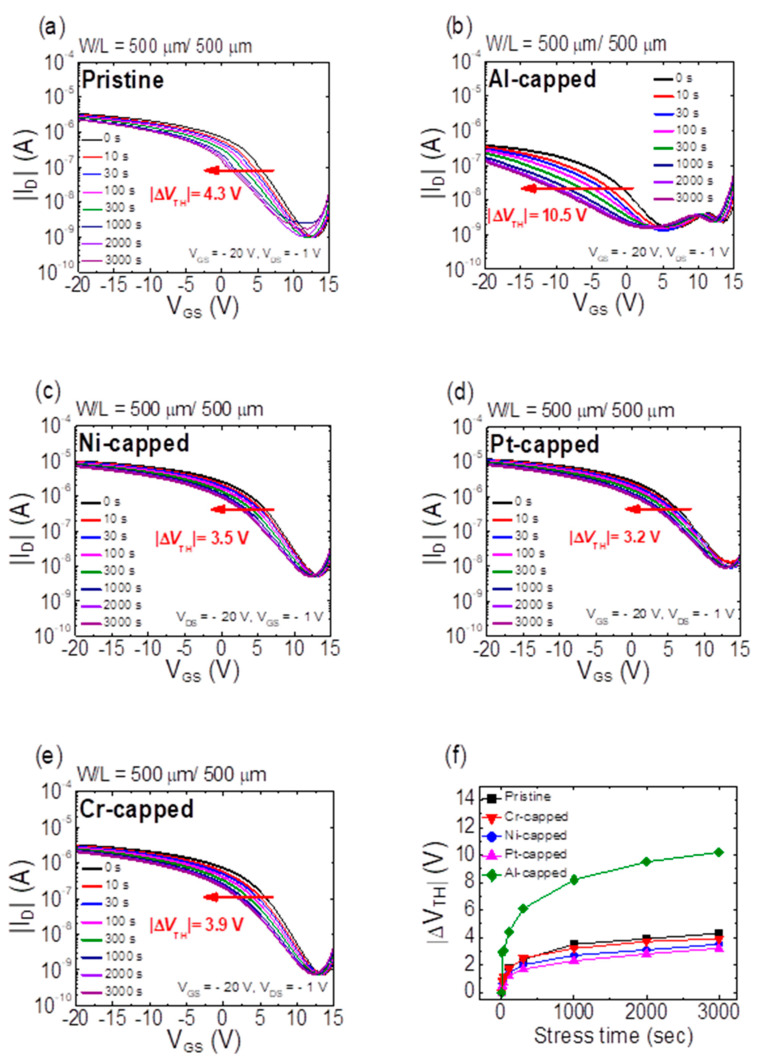
Time-dependent transfer curves of (**a**) pristine, (**b**) Al-capped, (**c**) Ni-capped, (**d**) Pt-capped, and (**e**) Cr-capped SnO TFTs under an application of constant bias stress of *V*_GS_ = −20 V and *V*_DS_ = −1 V. (**f**) Summary of Δ*V*_TH_ after a 3000 s stress in SnO TFTs with every metal capping layer.

Figure 7a–e present the time-dependent transfer curves of SnO TFTs with different capping metals under an application of constant bias stress of *V*_GS_ = −20 V and *V*_DS_ = −1 V. Figure 7f summarizes the *V*_TH_ shift (Δ*V*_TH_) after 3000 s of stress. Transfer curves shift in the negative direction with an increase in the stress time in all TFTs. The largest movement of *V*_TH_ is observed in the Al-capped SnO TFT. However, the Ni- and Pt-capped SnO TFTs exhibit smaller values of Δ*V*_TH_ than the pristine device after inducing negative-gate-bias-stresses (NGBSs). Figure 5 and Table 1 indicate that *SS* of the Al-capped SnO TFT exhibits the largest value; however, the Ni- and Pt-capped SnO TFTs exhibit smaller values of *SS* than the pristine SnO TFT. Considering that the negative-bias-stress-induced Δ*V*_TH_ and *SS* values significantly depend on the density of trap states located in the current-flow path, the obtained results in Figure 7 are consistent with the mechanisms suggested to explain the change of transfer curves by the different species of capping metals in Figure 5.

**Figure 8:**
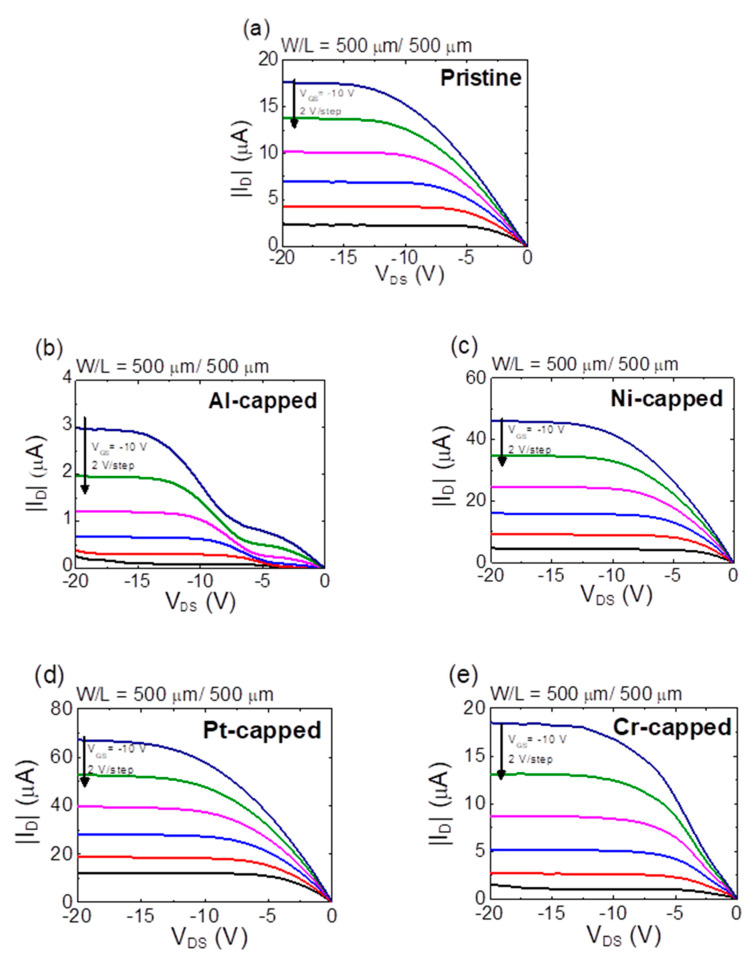
Output curves measured from the (**a**) pristine, (**b**) Al-capped, (**c**) Ni-capped, (**d**) Pt-capped, and (**e**) Cr-capped SnO TFTs.

Figure 8a–e display the output curves measured from the SnO TFTs with different capping metals. Figure 8 indicates that the output curves obtained from the pristine, Ni-capped, and Pt-capped SnO TFTs exhibit an excellent behavior with a clear pinch-off. However, severely distorted output curves were measured from the Al-capped SnO TFT, which can be attributed to the impeded carrier transport due to the additional energy barrier induced via the depletion layer formed by the Al capping layer [49]. Moreover, the slight current crowding phenomenon from the output curve measured from the Cr-capped SnO TFT can be observed, which indicates the probability of a shallow depletion region formed below the capping layer in the Cr-capped SnO TFT.

## 4. Conclusions

In this study, the electrical performance and stability of p-channel SnO TFTs containing capping layers of different metals (Al, Ni, Pt, and Cr) were compared. The SnO TFTs with a Ni or Pt capping layer showed an increased value of *μ*_FE_, a decreased value of *SS*, a positively shifted *V*_TH_, and an enhanced NGBS stability, compared to the pristine SnO TFT. In contrast, the SnO TFT with an Al capping layer showed a decreased value of *μ*_FE_, an increased value of *SS*, a negatively shifted *V*_TH_, and a deteriorated NGBS stability, compared to the pristine device. The SnO TFT with a Cr capping layer showed similar electrical performance and stability to the pristine SnO TFT. These results were majorly attributed to the different *Φ* values of the metals. The *Φ* of Al is lower than that of SnO, which causes the downward bend in the energy band and forms the depletion layer near the back-channel surface in SnO. The decreased hole concentration shifts *V*_TH_ in the negative direction and decreases the percolation conduction probability and *μ*_FE_. Contrary to the pristine TFT, the depletion region formed near the back-channel surface in the Al-capped SnO forces the holes to move closer to the SiO_2_/SnO interface, thereby degrading the *μ*_FE_, *SS*, and NGBS stability of the device. The *Φ* of Ni and Pt is higher than that of SnO, causing the upward energy band bending and forming the hole accumulation layer near the back-channel surface in the SnO. The increased hole concentration shifts *V*_TH_ in the positive direction and increases the percolation conduction probability and *μ*_FE_. In addition, the upward band bending forms the bulk accumulation channel during an application of the negative *V*_GS_ owing to the thickness of the thin film, which enhances the *μ*_FE_, *SS*, and NGBS stability of the device. Our experimental results show that the electrical property and stability of p-channel SnO TFTs significantly change depending on the types of metal capping layers. This demonstrates that the metal capping layer can be practically used to modulate the electrical characteristics of p-channel SnO TFTs.

## Figures and Tables

**Table 1:** Electrical parameters extracted from the SnO TFTs with different metal capping layers.

Parameter	Pristine	Al	Ni	Pt	Cr
*μ*_FE_ (cm^2^/V·s)	1.6	0.3	4.8	5.4	1.8
*SS* (V/dec)	4.0	4.7	3.7	3.5	3.9
*I*_ON_/_OFF_	2.5 × 10^3^	2.4 × 10^2^	1.8 × 10^3^	1.9 × 10^3^	2.7 × 10^3^
*V*_TH_ (V)	3.7	−1.6	5.2	6.3	4.5
